# Shunt performance in 349 patients with hydrocephalus after aneurysmal subarachnoid hemorrhage

**DOI:** 10.1007/s00701-021-04877-1

**Published:** 2021-06-24

**Authors:** Joona Tervonen, Hadie Adams, Antti Lindgren, Antti-Pekka Elomaa, Olli-Pekka Kämäräinen, Virve Kärkkäinen, Mikael von und zu Fraunberg, Jukka Huttunen, Timo Koivisto, Juha E. Jääskeläinen, Ville Leinonen, Terhi J. Huuskonen

**Affiliations:** 1 Department of Neurosurgery, Neurosurgery of KUH NeuroCenter, Kuopio University Hospital, Kuopio, Finland; 2 Faculty of Health Sciences, School of Medicine, Institute of Clinical Medicine, University of Eastern Finland, Kuopio, Finland

**Keywords:** Aneurysmal subarachnoid hemorrhage, Hydrocephalus, Critical care, Prognosis, Shunting, Revisions

## Abstract

**Background:**

Shunt-dependent hydrocephalus after aneurysmal subarachnoid hemorrhage (aSAH) is a common sequelae leading to poorer neurological outcomes and predisposing to various complications.

**Methods:**

A total of 2191 consecutive patients with aSAH were acutely admitted to the Neurointensive Care at the Kuopio University Hospital between 1990 and 2018 from a defined population. A total of 349 (16%) aSAH patients received a ventriculoperitoneal shunt, 101 with an adjustable valve (2012–2018), 232 with a fixed pressure valve (1990–2011), and 16 a valveless shunt (2010–2013). Clinical timelines were reconstructed from the hospital records and nationwide registries until death (n = 120) or June 2019.

**Results:**

Comparing the adjustable valves vs. the fixed pressure valves vs. the valveless shunts, intraventricular hemorrhage was present in 61%, 44% and 100%, respectively. The median times to the shunt were 7 days vs. 38 days vs. 10 days. The rates of the first revision were 25% vs. 32% vs. 69%. The causes included infection in 11% vs. 7% vs. 25% and overdrainage in 1% vs. 4% vs. 31%. The valveless shunt was the only independent risk factor (HR 2.9) for revision. After the first revision, more revisions were required in 48% vs. 52% vs. 45%.

**Conclusions:**

The protocol to shunt evolved over time to favor earlier shunt. In post-aSAH hydrocephalus, adjustable valve shunts, without anti-siphon device, can be installed at an early phase after aSAH, in spite of intraventricular blood, with a modest risk (25%) of revision. Valveless shunts are not recommendable due to high risk of revisions.

## Introduction

Aneurysmal subarachnoid hemorrhage (aSAH) is the third most common form of stroke (7 per 100,000)[12] at the lowest median age [21, 31, 35]. Acute aSAH is a complex systemic condition, requiring acute CT diagnosis and neurointensive care [7, 9, 31, 34]. Arterial bleed may cause an intracerebral (ICH) and/or intraventricular hematoma (IVH). Age, clinical condition, ICH, IVH, acute hydrocephalus, and extraventricular drainage (EVD) predict high mortality at 14 days [17], excess mortality at 12 months [19], and subsequent neurological outcome [13, 14, 16, 29].

Chronic hydrocephalus, requiring a permanent shunt, is frequent among aSAH survivors [1, 8, 36]. In our 1551 aSAH patients, six groups were identified with increasing risk (1% to 61%) of shunting [1]. Acute hydrocephalus, with or without IVH, predicted that EVD would be continued by a shunt, especially in case of bacterial infection [1, 10]. Normal ventricular volume on admission (56%) carried a notable risk (8%) of latent shunt [1]. Shunt failures, particularly infection and occlusion, requiring repeated revisions, are still frequent after decades of development [25]. Valveless shunts were suggested to reduce occlusion [2]. Adjustable valves have reduced overdrainage and the need for surgical revision [28]. Concerning shunted aSAH survivors, we found few long-term follow-up studies on shunt revisions [3, 27, 30].

A total of 2191 consecutive aSAH patients were admitted to the Kuopio University Hospital from a defined population (I/1990 to VI/2018). The timelines of the 349 patients with a ventriculoperitoneal shunt were analyzed until death or June 2019. The focus was the 101 patients with an adjustable valve (2012–2018), as compared to the 232 patients with a fixed pressure valve (1990–2011) and the 16 patients with a valveless shunt (2010–2013). The aim was to identify risk factors for the first shunt revision.

## Methods

### Literature review

PubMed was searched for English articles on humans published between January 2000 and November 2019 using the following terms: [(subarachnoid hemorrhage) OR SAH OR aSAH) AND (shunt* OR shunted OR shunting) AND (revision* OR complication* OR obstruction* OR infection*)]. We searched for the original articles on the aSAH patient cohorts that studied the shunted patients and the revisions of their shunts. Table 1 summarizes the cohorts regarded as relevant for the present study.

**Table 1:** Characteristics of previous relevant cohorts on shunt revisions in patients with shunt-dependent hydrocephalus after aneurysmal subarachnoid hemorrhage (aSAH)

Authors	Total aSAH patients (years)	Shunted patients (%)	First revisions (%)	Follow-up time	Shunt complications (%)	Risk factors for revision
O’Kelly et al., 2009^20^	3120 (1995–2004)	585 (19%)	173 (30%)	Mean 4.25 years	N/A	Posterior circulation sIA Endovascular treatment
Chalouhi et al., 2014^19^	N/A (2005–2010)	523 (N/A)	66 (13%)	Mean 2.0 years	Infection (n = 33; 6.3%) Ventricular catheter or valve failure (n = 32; 6.1%) ICH (n = 1; 0.2%)	Clipping of sIA and higher H&H for infection Clipping of sIA for proximal ventriculoperitoneal shunt revision
Paisan et al., 2018^21^	888 (2000–2015)	116 (13%)	21 (18%)	Mean 1.5 years	Infection (n = 8; 6.9%) Valve failure (n = 11; 9.5%)	WFNS ≥ III Posterior circulation sIA Vasospasm
Tervonen et al., 2021	2191 (1990–2018)	349 (16%) 232 fixed pressure valves 16 valveless shunts 101 adjustable valves	110 (32%)	Median 8.3 years (IQR 3.6–15 years)	Infection (n = 31; 8.9%) Valve occlusion (n = 35; 10%) Ventricular catheter malposition or occlusion (n = 21; 6.0%) Peritoneal catheter occlusion (n = 5; 1.4%)	Valveless shunt; HR = 2.94

Abbreviations: *N/A*, not applicable; *WFNS*, World Federation of Neurological Surgeons SAH grade (I–V); *H&H*, Hunt and Hess grade (I–V); *sIA*, saccular intracranial aneurysm; *EVD*, extraventricular drainage; *ICH*, intracerebral hemorrhage

### Catchment population of Kuopio University Hospital (KUH)

KUH, one of the five University Hospitals in Finland, is an academic, non-profit, publicly funded tertiary center, which has solely provided full-time acute and elective neurosurgical services for the defined KUH catchment population of approximately 850,000 in Eastern Finland. The KUH area contains four Central Hospitals, each with 24/7 neuroacutology, CT services, and intensive care [17]. During the study period from 1990 to 2018, all cases of SAH (CT and spinal tap) were acutely transferred to KUH for neurointensive care, neuroradiology (4-vessel angiography and/or CT angiography), and neurosurgery [17]. Neurointensive care was provided virtually regardless of the age or condition on admission, including H&H 4–5 patients. A dedicated team of neurointensivists, neurosurgeons, and neuroradiologists coordinated the aSAH treatment. KUH Neurovascular Group evacuated significant ICHs with immediate microsurgery. The KUH aSAH protocol followed international recommendations in detail [7, 9, 31, 34], including routine extraventricular drainage (EVD) and parenchymal ICP monitoring, decompressive craniectomy (DC) when needed, and intra-arterial nimodipine in delayed brain ischemia.

### Kuopio Intracranial Aneurysm (IA) Patient and Family Database

The database, prospective since 1995, contains all cases of unruptured and ruptured IAs admitted to KUH since 1980. A dedicated, full-time nurse administrates the database; interviews all new IA patients, including their family history; and arranges the follow-ups. The clinical data, including prescribed medicines, hospital diagnosis, and causes of death, have been fused from the national registries, using the Finnish personal codes. We have previously characterized the aSAH patients, e.g., diabetes mellitus [24], hypertension [23], for the 14-day mortality and organ donation [17], 12-month mortality [15], shunt-dependent hydrocephalus [1], epilepsy [14], psychosis [13], and long-term excess mortality [16]. Three 1-degree relatives with a diagnosed sIA disease form an sIA family.

### Study population

**Fig. 1:**
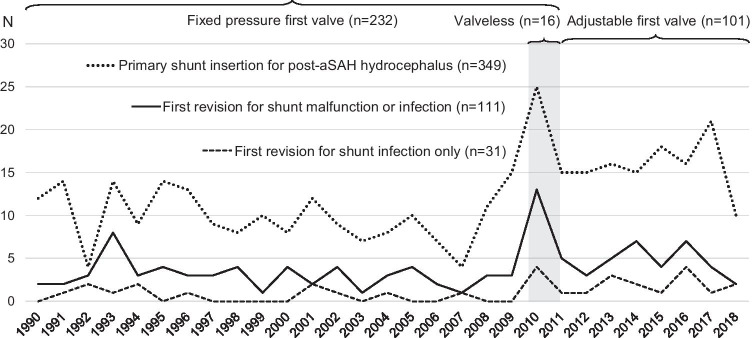
Timelines of three shunt types in 349 patients with hydrocephalus after aneurysmal subarachnoid hemorrhage (aSAH). A total of 2191 consecutive patients with aSAH from ruptured saccular intracranial aneurysm (sIA) were acutely admitted to the Neurointensive Care at the Kuopio University Hospital (KUH) between 1990 and 2018 from its defined Eastern Finnish catchment population. The study cohort consists of 349 consecutive aSAH patients with a primary ventriculoperitoneal shunt inserted for post-aSAH hydrocephalus. The annual numbers of the primary shunt insertions (n = 349) and their first revisions (n = 111) for either malfunction (n = 80) or shunt infection (n = 31) are indicated. Altogether, 232 fixed pressure shunt valves were primarily inserted from 1990 to 2011, and thereafter, only adjustable shunt valves (n = 101) were primarily used. The short period when most of the valveless shunts (n = 16) were inserted is indicated

A total of 2191 consecutive adult (> 18 years) aSAH patients were acutely admitted to the KUH Neurointensive Care from 1990 to 2018 from a defined population (Fig. 1). Their clinical lifelines have been reconstructed from their clinical data in the Kuopio database and from the national clinical registries until death or the last follow-up [13, 29]. The final study population consisted 349 aSAH patients who received their first ventriculoperitoneal shunt in a median time of 24 days (IQR 9–60 days) after aSAH (Fig. 1, Table 2).

**Table 2:** The study population of 349 consecutive aSAH patients who received their first ventriculoperitoneal shunt with three different valve types after aSAH. They had been acutely admitted to the Neurointensive Care of Kuopio University Hospital from its Eastern Finnish catchment population from 1990 to 2018

	Adjustable valve 2012–2018 n = 101	Valveless shunt 2010–2013 n = 16	Fixed pressure valve 1990–2011 n = 232
Females	69 (62%)	12 (75%)	130 (56%)
Member of sIA family	1 (1%)^2^	0	24 (10%)^2^
Median age at aSAH (years, IQR)	59 (50–68)	56 (47–63)	58 (47–66)
Site of ruptured sIA			
Internal carotid artery Anterior carotid artery Middle cerebral artery Vertebrobasilar artery	23 (23%) 42 (42%) 21 (21%) 15 (15%)	7 (44%) 5 (31%) 2 (13%) 2 (13%)	44 (19%) 108 (47%) 43 (19%) 37 (16%)
Median size of ruptured sIA (mm, IQR)	6 (4–9)^2^	6 (4–9)	7 (5–10)^2^
Multiple sIAs (> 2)	22 (22%)	6 (38%)	71 (31%)
Hunt and Hess grade on admission			
I II III IV V	14 (14%) 22 (22%) 23 (23%) 33 (33%) 9 (9%)	1 (6%) 3 (19%) 2 (13%) 9 (56%) 1 (6%)	21 (9%) 56 (24%) 88 (38%) 55 (24%) 12 (5%)
Intracerebral hemorrhage	27 (27%)	4 (25%)	59 (25%)
Intraventricular hemorrhage	61 (61%)^1,2^	16 (100%)^1,3^	102 (44%)^2,3^
Extraventricular drainage	82 (82%)^2^	16 (100%)^3^	156 (67%)^2,3^
Meningitis prior shunt	12 (12%)	3 (19%)	46 (20%)
Median days to first shunt (days, IQR)	7 (5–16)	10 (8–14)	38 (21–90)
Antibiotic-impregnated shunt Silver-impregnated shunt*	65 (65%)^2^ 21 (21%)	16 (100%)^3^ n.r	20 (9%)^2,3^ 0 (0%)
First revision Median time to first revision (days) Two or more revisions per patient	25 (25%) 7 (4–26) 12 (12%)	11 (69%) 17 (9–104) 5 (31%)	75 (32%) 48 (7–181) 39 (17%)
Median revision-free time after shunt (months)	47 (25–73)	72 (9–92)	146 (92–232)
Deaths during follow-up	15 (15%)	4 (25%)	105 (45%)

Abbreviations: IQR, interquartile range; sIA, saccular intracranial aneurysm; aSAH, subarachnoid hemorrhage from ruptured sIA. *The proximal catheter silver-impregnated, the distal catheter antibiotic-impregnated

The superscript indicates the statistical significance (Kruskal–Wallis, p < 0.05) as follows: 1, adjustable vs. valveless; 2, adjustable vs. fixed; 3, valveless vs. fixed

### Variables and clinical data retrieval sources

The characteristics and variables of the patients (n = 349) and the primary shunts are described in Table 2. The clinical data was retrieved and reviewed from the following sources: Kuopio IA Database, with data fusion from the national registries; KUH case reports, referrals from the four Central Hospitals, and neurosurgical operative reports on shunt installations and revisions (manually 1990–2004; digitally until June 2019); neuroimaging (manually 1990–2002; digitally until June 2019); monitoring data before, during, and after all shunt installations and revisions; and laboratory and bacteriology data. The follow-up time ended at death (n = 120) or June 2019. No patients were lost to follow-up.

### Shunt surgery and follow-up for shunt complications

In the KUH catchment population, all adult shunt installations and revisions have been performed by KUH Neurosurgery. The shunted patients have been followed by the KUH Neurosurgery and Neurology Departments of the four Central Hospitals. During the study period since 1990, all Hospitals have had 24/7 CT services. Since 2010, adjustable valves have been reset also in the Central Hospitals. KUH Neurosurgery have treated all shunt complications, e.g., occlusions, infections, intracranial hematomas, overdrainages (clinical and/or radiological signs leading to valve adjustment or shunt revision), and subdural effusions.

### Types of shunt valves used in 349 aSAH patients

During the recruitment period from 1990 to 2018, three types of valves were used (Table 2, Fig. 1). The adjustable valves without anti-siphon device enabled us to insert shunts earlier thus reducing the time of EVD.

1. Fixed pressure valves (n = 232):
  Exclusively from 1990 to 2009 (n = 198); occasionally from 2010 to 2017 (n = 34)
   > 5 mmHg opening pressure (n = 196)
  0–5 mmHg opening pressure (n = 36)
2. Adjustable valves (n = 101):
  Used since 2010 and the main valve type since 2012.
  Medtronic Strata NSC without an anti-siphon device (n = 86) or Medtronic Strata II (n = 15). Valve without anti-siphon device was used to reduce the risk for obstruction due to blood in CSF.
  Control head CT (n = 97) in a median of 3 days (IQR 2–6), MRI (n = 3), or skull x-ray (n = 1) to verify the ventricular catheter position after the shunt installation.
3. Valveless primary shunts in the study were Codman Uni-Shunts (n = 16); these were inserted between 2010 and 2013.

Antibiotic prophylaxis (cefuroxime) was routinely administered i.v. (i) during the EVD and shunt installations (1.5 g) and (ii) administered i.v. 1.5 g three times a day during the EVD period.

### Statistical analysis

Continuous variables were reported as medians and interquartile ranges (IQR). Frequencies and percentages were used with the categorical and the dichotomous variables. The association of the continuous variables on the first shunt revision was analyzed with the Mann–Whitney U test or the Kruskal–Wallis test. Categorical variables were evaluated by the χ2 test. The Cox regression analysis was used to identify the independent risk factors for the first shunt revision. Statistical analysis was performed using SPSS version 25.0 (SPSS, Inc., Chicago, IL). The differences were statistically significant if the p-value was < 0.05.

### Ethical approvals

The study was approved by the Ethical Committee of the Kuopio University Hospital. This was a retrospective database study including only the patients already treated in KUH for aSAH. The need for additional consent for this registry study was waived by the Ethics Committee of KUH. The data integration from the national registries was performed with the approval of the Ministry of Social Affairs and Health of Finland and the National Institute for Health and Welfare.

## Results

**Fig. 2:**
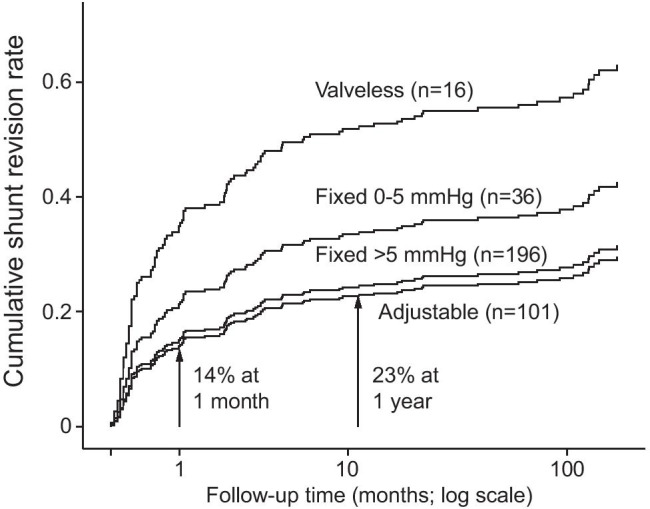
Cumulative rates of first revision in 349 aSAH patients with different shunt types. The cumulative rate of the first revision of the 349 consecutive aSAH patients with a primary ventriculoperitoneal shunt inserted for post-aSAH hydrocephalus using Cox regression analysis. The follow-up time is logarithmic to emphasize the first months. The revision rate of the 101 patients with an adjustable valve at 1 month and at 1 year is presented (arrows)

The study population consisted of 349 aSAH patients who received their first ventriculoperitoneal shunt from 1990 to 2018 with three almost consecutive valve types after aSAH (Fig. 1, Table 2). Of the 349 patients, 111 (32%) underwent the first shunt revision in median of 23 days (IQR 6–111) until death (n = 124) or June 2019, a total of 2513 follow-up years and a median of 8.3 years (Fig. 2).

### Overall risk of shunt revision

The risk for the first revision was equal for the 101 adjustable valve shunts and the 196 fixed medium-pressure valve shunts but significantly lower (p = 0.01) than for the 16 valveless shunts (Table 2, Fig. 2). In multivariate Cox regression analysis, the valveless shunt was the only independent risk factor (HR 2.9), with age group ≤ 57 years, EVD, and fixed low-pressure valve showing no significance (Table 3). Hunt and Hess grade, antibiotic-impregnated catheters, ICH, and IVH were tested but did not have statistical significance in the multivariate analysis.

**Table 3:** Univariate and multivariate Cox regression analyses for shunt revision risk

Factor	Patients (n = 349)	Hazard ratio	Univariate 95% CI	p-value	Hazard ratio	Multivariate 95% CI	p-value
Age > 57 years	184 (53%)	1.00		Ref	1.00		Ref
Age ≤ 57 years	165 (47%)	1.44	0.97–2.10	0.059	1.34	0.92–1.97	0.131
No EVD	95 (27%)	1.00		Ref	1.00		Ref
EVD	254 (73%)	1.57	0.99–2.48	0.055	1.37	0.85–2.21	0.198
Programmable valve	101 (29%)	1.00		Ref	1.00		Ref
Valveless shunt	16 (5%)	3.19	1.53–6.66	0.002*	2.94	1.40–6.15	0.004*
Fixed pressure valve 0–5 mmHg	36 (10%)	1.62	0.84–3.12	0.149	1.61	0.84–3.10	0.167
Fixed pressure valve > 5 mmHg	196 (56%)	1.06	0.66–1.70	0.818	1.12	0.69–1.81	0.719

Abbreviations: *EVD*, extraventricular drainage; *Ref*, reference in logistic regression analysis

^*^Statistically significant p-values < 0.05

### Adjustable valves (n = 101; 2012–2018)

**Fig. 3:**
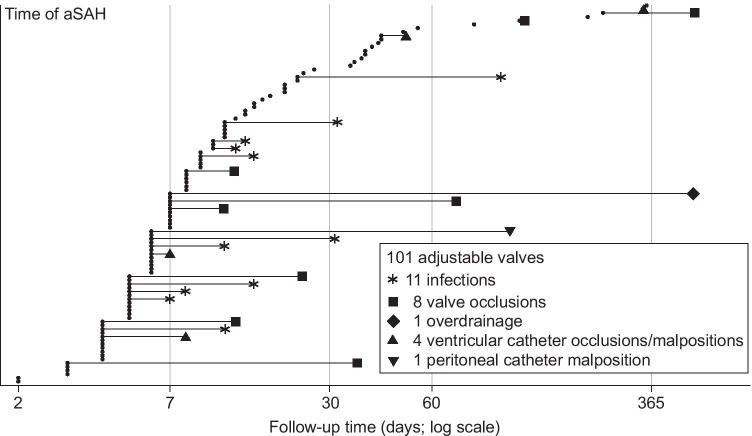
Clinical timelines of 101 aSAH patients with adjustable valve shunt. The 101 consecutive aSAH patients with a primary ventriculoperitoneal shunt (adjustable valve) inserted for post-aSAH hydrocephalus from 2010 to 2018 (see Table 2 and Fig. 4). The follow-up time is logarithmic. For each patient, the time in days (median 7 days; IQR 5–16 days) from aSAH to the first shunt insertion (black dot) is arranged according to the increasing length of the time interval. The time in days (median 7 days; IQR 4–26 days) from the shunt insertion (black dot) to the first revision (symbols) in 25 (25%) of the 101 patients is presented with a thin horizontal line according to increasing length of the revision-free time

**Fig. 4:**
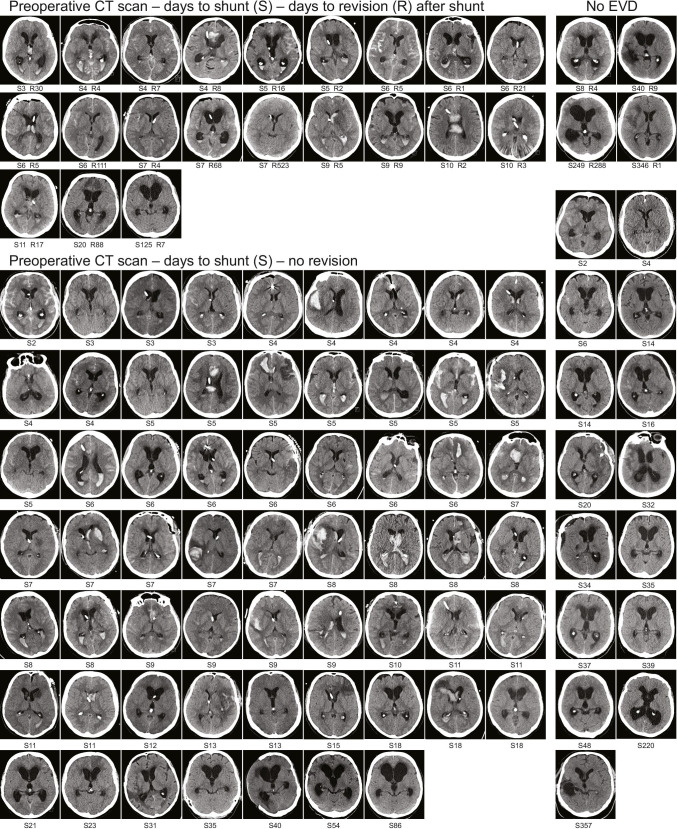
Representative CT slices of 101 aSAH patients before shunt insertion. The 101 consecutive aSAH patients with a ventriculoperitoneal shunt (adjustable valve) inserted from 2010 to 2018 (Table 2 and Fig. 3). The figure shows one representative CT slice from the last available imaging of each patient before the shunt insertion. Under each CT slice are the days to the shunt insertion (S) since aSAH. A total of 25 patients had a shunt revision (three upper rows) with the days to the shunt revision (R) under their CT slices. EVD (extraventricular drainage) was installed in 82 patients (nine left columns). Due to EVD or spinal drainage, some ventricles are small at the time of the shunt insertion, and in some cases, EVD is removed and followed by spinal drainage

Of the 349 shunted patients, 101 had an adjustable valve (Table 2, Fig. 3). Figure 4 presents a representative CT slice of each patient before the shunt insertion, with a special emphasis on the intraventricular blood amount. The puncture sites were frontal (n = 94) or occipital (n = 7).

Antibiotic-impregnated shunt was used in 65 patients and silver-impregnated ventricular catheter with antibiotic-impregnated distal catheter in 21 patients (Table 4).

**Table 4:** The study cohort of 349 consecutive aneurysmal subarachnoid hemorrhage (aSAH) patients with a primary ventriculoperitoneal shunt due to post-aSAH hydrocephalus from 1990 to 2018

	Adjustable valves 2012–2018 n = 101	Valveless shunts 2010–2013 n = 16	Fixed pressure shunts 1990–2011 n = 232
Median time to shunt (days)	7 (5–16)^2^	10 (8–14)^3^	38 (21–90)^2,3^
Median follow-up after shunt (months)	47 (25–66)^2^	90 (19–106)^3^	133 (91–227)^2,3^
First revision Median time to first revision (days)	25 (25%)^1^ 7 (4–26)^2^	11 (69%)^1,3^ 17 (9–104)	75 (32%)^3^ 48 (7–181)^2^
Shunt infection Median time to revision (days) Antibiotic-impregnated shunt Silver-impregnated shunt* Normal catheter	11 (11%) 5 (3–17) 8/65 3/21 0/15	4 (25%) 12 (7–16) 4/16 n.r n.r	16 (7%) 96 (12–513) 1/20 0/0 15/212
Valve occlusion Median time to revision (days)	8 (8%) 12 (5–59)	n.r	27 (12%) 33 (7–198)
Ventricular catheter malposition Median time to revision (days)	1 (1%)	0	11 (5%) 6 (3–70)
Ventricular catheter occlusion Median time to revision (days)	3 (3%) 4 (3–6)	2 (13%)	4 (2%) 7 (5–13)
Peritoneal catheter occlusion/malposition Median time to revision (days)	1 (1%)	0	4 (2%) 65 (7–113)
Catheter disconnection	0	0	3 (1%)
Shunt overdrainage leading to revision Median time to revision (days)	1 (1%)^1^	5 (31%)^1,3^ 30 (17–104)	10 (4%)^3^ 90 (57–443)

^*^The proximal catheter silver-impregnated, the distal catheter antibiotic-impregnated

The superscript indicates the statistical significance (Kruskal–Wallis, p < 0.05) as follows: 1, adjustable vs. valveless; 2, adjustable vs. fixed; 3, valveless vs. fixed

The initial valve settings were either 1.5 T (n = 78), 1.0 T (n = 19), 0.5 T (n = 2), or 2.0 T (n = 2). One or more adjustments were required in 32 (32%) of the 101 patients, due to clinical overdrainage in 17 (17%) in median of 71 days (IQR 6–129) and clinical underdrainage in 15 (15%) in a median of 4 days (IQR 3–14).

Of the 101 patients, 82 (81%) had EVD and 19 (19%) had not (Fig. 3). The median time to shunt insertion was 7 days (IQR 5–11) with EVD (acute hydrocephalus) and 34 days (IQR 14–48) without EVD (latent hydrocephalus). The median follow-up time since shunt insertion, until death (n = 15) or June 2019, was 47 months (IQR 25–66).

No shunt revision was required in 76 (75%) of the 101 patients, until death (n = 10) or June 2019, a median revision-free time of 47 months. Of these 76 patients though, 23 (30%) required adjustments of the valve settings, at least once. In overall, 53 (52%) of the 101 patients, with a median time of 8 days to shunt insertion, did not require any shunt revisions or adjustments (Fig. 3).

Shunt revision was required in 25 (25%) of the 101 patients, until death (n = 5) or June 2019, in a median time of 7 days since shunt insertion (Table 4, Fig. 3). The cumulative rate for the first revision was 14% at 1 month and 23% at 1 year (Fig. 2). Of the 25 patients with shunt revision, nine had previous valve adjustments. In overall, multiple (two or more) revisions were required in 12 (48%) of the 25 revised patients.

Shunt infection (n = 11) was the most common cause for shunt revision, in a median of 5 days (Table 4, Fig. 3). All 11 patients had EVD and i.v. cefuroxime prophylaxis since the shunt installation. Ten patients had *clinical meningitis*, including decreased CSF glucose, and one had a wound infection. However, bacterial cultures remained negative, except in two cases (*Staphylococcus warneri* and *Staphylococcus aureus*) considered possible contaminations. The PCR testing of CSF samples was not performed. The infections somewhat clustered in the early shunts (Fig. 3).

Valve occlusion (n = 8) was the second most common reason for shunt revision, in a median of 12 days (Table 4, Fig. 3). The 15 shunts with the anti-siphon device were installed in a median of 39 days (IQR 8–220), and only one of them occluded. The 86 shunts without the anti-siphon device were installed much earlier (see Methods), in a median of 7 days (IQR 5–11), and seven of them occluded.

### Valveless shunts (n = 16; 2010–2013)

Of the 349 shunted patients, 16 (4.6%) had a valveless shunt in a median of 10 days after aSAH (Table 2, Table 4, Fig. 1). All 16 patients had EVD. The median follow-up time since shunt insertion, until death (n = 4) or June 2019, was 90 months (19–106).

No shunt revision was required in five (31%) patients, until death (n = 3) or June 2019, a median revision-free time of 72 months. Shunt revision was required in 11 (69%) patients, until death (n = 1) or June 2019, in a median of 17 days (Table 4, Fig. 2). The causes of revision were shunt overdrainage (n = 5), shunt infection (n = 4), and ventricular catheter occlusion (n = 2) (Table 4). In overall, multiple revisions were required in 5 (45%) of the 11 revised patients.

### Fixed pressure valves (n = 232; 1990–2011)

Of the 349 shunted patients, 232 (66%) had a fixed pressure valve, 36 with a low-pressure valve (0–5 mmHg), and 196 with a medium-pressure valve (> 5 mmHg). Of the 232 patients, 156 (67%) had EVD and 76 (33%) had not. The median time to shunt insertion was 35 days (IQR 16–86) with EVD (acute hydrocephalus) and 48 days (IQR 24–100) without EVD (latent hydrocephalus). The median follow-up time since shunt insertion, until death (n = 105) or June 2019, was 133 months (91–227).

No shunt revision was required in 157 (68%) patients, until death (n = 72) or June 2019, a median revision-free time of 146 months (Table 2). Shunt revision was required in 75 (32%) patients, until death (n = 33) or June 2019, in a median of 48 days (Table 2, Fig. 2). The two most common causes of revision were valve occlusion (n = 27) and shunt infection (n = 16), reversal as compared to adjustable valves (Table 4). In overall, multiple revisions were required in 39 (52%) of the 75 revised patients.

### Risk of revision with adjustable valve, fixed pressure valve, and valveless shunts

As Table 3 and Fig. 2 indicate, the risk for the first revision was equal for the 101 adjustable valve shunts and the 196 fixed medium-pressure valve shunts but significantly lower (p = 0.01) than for the 16 valveless shunts.

## Discussion

In our basic cohort of 2191 consecutive aSAH patients from a defined population (1990–2018), a total of 349 (17%) required a permanent ventriculoperitoneal shunt due to acute or latent hydrocephalus. In two previous large cohorts, the shunt dependency was 13% and 19% (Table 1) in 888 and 3120 aSAH patients, respectively [27, 30]. Shunt failures in hydrocephalus of various causes, including aSAH, which may require repeated revisions, are frequent after 40 decades of development [25].

Shunt failures may be (i) mechanical (catheter disinsertion; catheter disconnection; exposure to skin), (ii) biological (meningitis; infection of the shunt; occlusion of catheters or valves), or (iii) hydrodynamic (underdrainage; overdrainage). We found few large long-term follow-up studies on the shunt revisions in post-aSAH hydrocephalus [3, 27, 30].

In the present study of 349 shunted patients, the clinical date point timelines, reconstructed with the personal identity codes from the Finnish nationwide clinical registries, were analyzed until death (n = 124) or June 2019 with none lost to follow-up. A total of 111 (32%) patients underwent at least one shunt revision. In multivariate analysis, no other independent risk factors for the shunt revision than the use of valveless shunt were found. The focus was the 101 patients with an adjustable valve (2012–2018), as compared to the 232 patients with a fixed pressure valve (1990–2011) and the 16 patients with a valveless shunt (2010–2013) (Fig. 1).

In the 101 aSAH patients with an adjustable valve, the median time from the admission to the shunt was 7 days (Figs. 3and 4). No shunt revision was required in 75%, a median revision-free time of 47 months. In 32 patients, the valve settings had to be adjusted after the shunt, and nine of them required a surgical shunt revision. In overall, shunt revision was required in 25% in a median of 7 days. The most common causes were shunt infection (11%) in a median of 5 days, in spite of intraoperative cefuroxime prophylaxis, and shunt occlusion (8%) in a median of 12 days. In the historic fixed pressure valve group (Fig. 1), the shunts were installed (median 38 days) and revised (median 48 days) significantly later, at an almost equal rate though (Fig. 2). In two previous aSAH cohorts, the adjustable valve shunts were less often revised as compared to the fixed pressure valve shunts: 7% and 9% vs. 22% and 30%, respectively [22, 28].

The adjustable valves, in our hands without the anti-siphon device, brought an important paradigm shift in our service as compared to the period of fixed pressure valves. Shunt valves without anti-siphon device enabled us to install shunts earlier to minimize the risk of occlusion and to reduce the time of EVD. Early shunt excludes the possibility to use EVD in maintaining an adequate CPP in case of severe vasospasm. However, early shunt was considered as soon as ICP monitoring was not required due to the decreased level of consciousness, but patients were still requiring CSF drainage. Early shunt can also shorten the neurointensive care period and can lead to earlier mobilization. Prolonged EVD or spinal drainage are risk factors for meningitis, even with antibiotic prophylaxis and use of antibiotic-impregnated catheters [10]. We can speculate that due to the use of antibiotic-impregnated catheters, the shunt infection rate was not increased despite of earlier shunt insertion. Two previous studies also supported early conversion of EVD into a ventriculoperitoneal shunt [18, 32]. Finally, adjustable valve shunts resolved the problems of overdrainage and underdrainage, previously requiring surgical revisions. This tallies with a recent study comparing 173 fixed pressure valves and 16 adjustable valves [6].

In our study, 16 primary valveless shunts were inserted in a brief 4-year period (Fig. 1), with the idea that shunting could take place at an earlier phase and with more blood in the CSF. However, the valveless shunts in our hands proved unsatisfactory: as many as 11 (69%) required at least one revision, five (31%) of them for overdrainage (Fig. 2). In contrast, in the Danish population-based cohort of 214 post-hemorrhagic hydrocephalus patients, including 161 SAH patients, there was no significant difference in the revision rates between the 137 valveless (26%) and 77 valve-regulated (29%) ventriculoperitoneal shunts [2]. Instead, the rate of overdrainage was significantly higher with the valveless shunts: 40% vs. 9%. In our overall series, the rates of overdrainage were 1%, 4%, and 31% for the 101 adjustable valve, 232 fixed pressure valve, and 16 valveless ventriculoperitoneal shunts, respectively. Our findings emphasize the need for clinical trials and prospective quality registries when implementing new innovations in neurosurgery.

The strengths of the present study are derived from the free tax-paid Finnish health care system. The five university hospitals of Finland have their own catchment areas which supports disease cohorts with minimal selection and long-term follow-ups with almost none dropped. Using the Finnish identity codes, all contacts to the healthcare system are automatically archived in the national clinical registries. Our follow-up was considerably longer as compared to previous studies (Table 1).

All shunt insertions and revisions were performed by the KUH neurosurgeons. There are also limitations. Our study is retrospective, although the database prospectively collected all aSAH patients through the study period. Furthermore, the detailed neurointensive care monitoring data, digitally available in the Finnish nationwide database [31], was not utilized here. Such data would be important for more detailed prediction of shunt occlusions, infections, and revisions in post-aSAH hydrocephalus.

The mechanisms, such as neuroinflammation, behind acute, latent, and chronic post-aSAH hydrocephalus are not fully understood, e.g., possible alterations in ependymal cells, ciliary beat, arachnoid villi, CSF production and resorption, or glymphatic circulation [11, 20]. Considering neuroinflammation, the use of dexamethasone after aSAH has been associated with the reduction in unfavorable outcome and the incidence of hydrocephalus [5, 26, 33]. The impact of dexamethasone, or any other modulator of inflammation, on the revision rates in shunted aSAH has not been established. Individual factors, including advanced age, concomitant diseases, and long neurointensive care, may affect the individual predisposition or resilience to shunt occlusions or infections — and might be modifiable.

Risk factors of shunt occlusion or shunt infection are difficult to define. There is no standard diagnosis for shunt infection, and different guidelines are used [37]. Negative CSF or catheter culture does not totally rule out shunt infection, especially under antibiotic prophylaxis, such as cefuroxime during our early conversions of EVD to shunts. Furthermore, asymptomatic infection may cause shunt occlusion. For further research, it is important to use PCR or sequencing of CSF, catheters, and valve contents to identify the causative pathogen or pathogens in all cases of occluded or infected shunt material.

Long-term outcome and quality of life of shunted aSAH survivors require further research. Risk factors of repeated revisions and possible methods to prevent them should be investigated. In our study, 12 (12%) of the 101 adjustable valve shunt patients and 39 (17%) of the 232 fixed pressure valves underwent two or more revisions. Among the 585 patients with a ventriculoperitoneal shunt for post-aSAH hydrocephalus, 74 (13%) underwent two or more revisions (32 at least three; 21 at least four) [27]. O’Kelly et al. [27] were unable to glean risk factors for the multiple shunt revisions. Finally, it is not known whether shunt-dependent hydrocephalus after aSAH is an independently dementing brain condition, apart from brain injuries at the acute and subacute phase [4].

## Conclusions

Our analysis of 349 patients with a ventriculoperitoneal shunt for hydrocephalus due to aneurysmal subarachnoid hemorrhage (aSAH) suggests the following:

- 1. The protocol to shunt evolved over time to favor earlier shunt instead of weaning.
- 2. Adjustable valve shunts, in our hands without the anti-siphon device, can be installed at an early phase after aSAH with a modest risk (25%) of shunt revision.
- 3. Valveless shunts are not recommendable due to significantly higher risk of revision as compared to shunts with adjustable opening pressure.
